# Therapeutic Potential and Biological Applications of Cordycepin and Metabolic Mechanisms in Cordycepin-Producing Fungi

**DOI:** 10.3390/molecules24122231

**Published:** 2019-06-14

**Authors:** Peng Qin, XiangKai Li, Hui Yang, Zhi-Ye Wang, DengXue Lu

**Affiliations:** 1 Institute of Biology, Gansu Academy of Sciences, Lanzhou 730000, Gansu, China; kingkerberos@163.com (P.Q.); yanghui43@163.com (H.Y.); Zhiye_wang@sina.com (Z.-Y.W.); 2 Ministry of Education Key Laboratory of Cell Activities and Stress Adaptations, School of Life Sciences, Lanzhou University, Lanzhou 730000, Gansu, China; xkli@lzu.edu.cn; 3 Key Laboratory of Microbial Resources Exploition and Application of Gansu Province, Institute of Biology, Gansu Academy of Sciences, Lanzhou 730000, Gansu, China

**Keywords:** cordycepin, medicinal targets, biological value, metabolic mechanisms

## Abstract

Cordycepin (3′-deoxyadenosine), a cytotoxic nucleoside analogue found in *Cordyceps militaris*, has attracted much attention due to its therapeutic potential and biological value. Cordycepin interacts with multiple medicinal targets associated with cancer, tumor, inflammation, oxidant, polyadenylation of mRNA, etc. The investigation of the medicinal drug actions supports the discovery of novel targets and the development of new drugs to enhance the therapeutic potency and reduce toxicity. Cordycepin may be of great value owing to its medicinal potential as an external drug, such as in cosmeceutical, traumatic, antalgic and muscle strain applications. In addition, the biological application of cordycepin, for example, as a ligand, has been used to uncover molecular structures. Notably, studies that investigated the metabolic mechanisms of cordycepin-producing fungi have yielded significant information related to the biosynthesis of high levels of cordycepin. Here, we summarized the medicinal targets, biological applications, cytotoxicity, delivery carriers, stability, and pros/cons of cordycepin in clinical applications, as well as described the metabolic mechanisms of cordycepin in cordycepin-producing fungi. We posit that new approaches, including single-cell analysis, have the potential to enhance medicinal potency and unravel all facets of metabolic mechanisms of cordycepin in *Cordyceps militaris*.

## 1. Introduction

**Figure 1:**
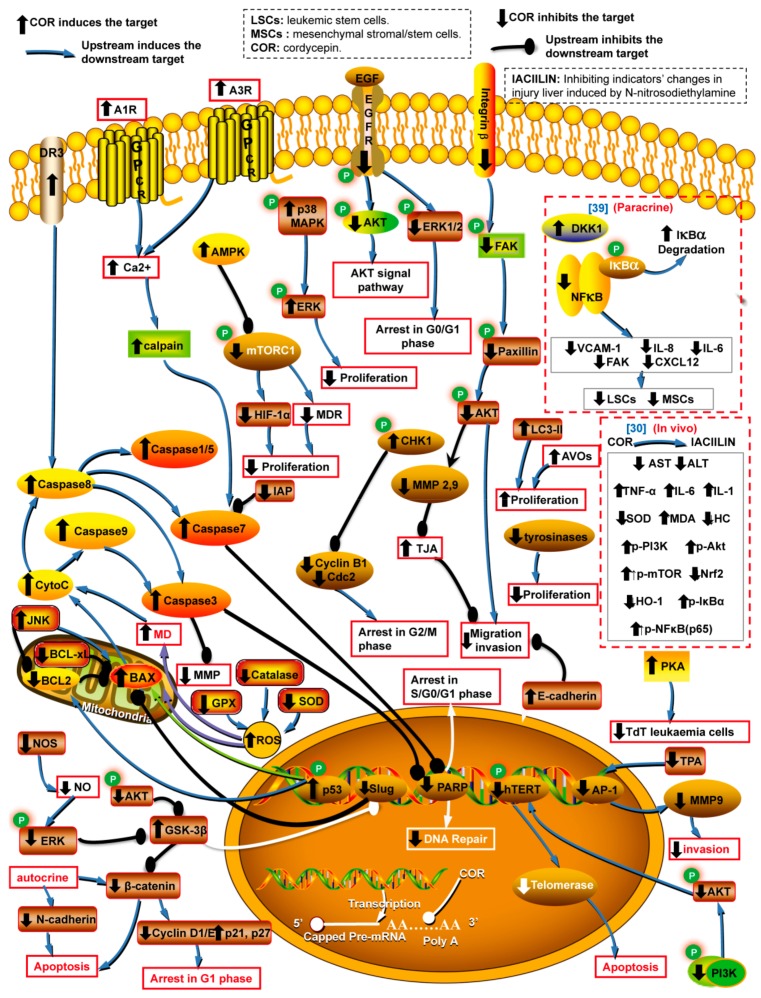
Cellular targets of cancer affected by cordycepin via various signal pathways. Note: A1R: adenosine A1 receptor. A3R: adenosine A3 receptor. GPCR: G protein-coupled receptor. TJA: tight junction activity. IL: interleukin. SOD: Superoxide Dismutase. NOS:nitric oxide synthase. CHK1: Checkpoint kinase 1. GPX: Glutathione peroxidase. EGFR: epidermal growth factor receptor. AVOs: acidic vesicular organelles. P-: phosphorylated. MMPs: matrix metalloproteinases, such as MMP-2 and MMP-9. AP-1 and NF-κB: transcription factors that bind to the promoter of MMP-9 gene and play an important role in regulating MMP-9 [15]. LC3-II: an autophagosome marker, and the cytoplasmic form LC3-I (18 kDa) is converted to LC3-II during autophagy [16]. DR3: death receptor3. TPA: 12-O-tetradecanoylpho-bol-13-acetate. PARP: Poly (adenosine-diphosphate-ribose) polymerase. HC: histopathology condition. HIF-1α: hypoxia-inducible factor 1α. MDR: multiple drug resistant. AMPK: adenosine 5′-monophosphate-activated protein kinase. MD: mitochondrial disfunction. The blue numbers in square brackets represent references associated with in vivo results. These abbreviations are also used in this paper.

**Figure 2:**
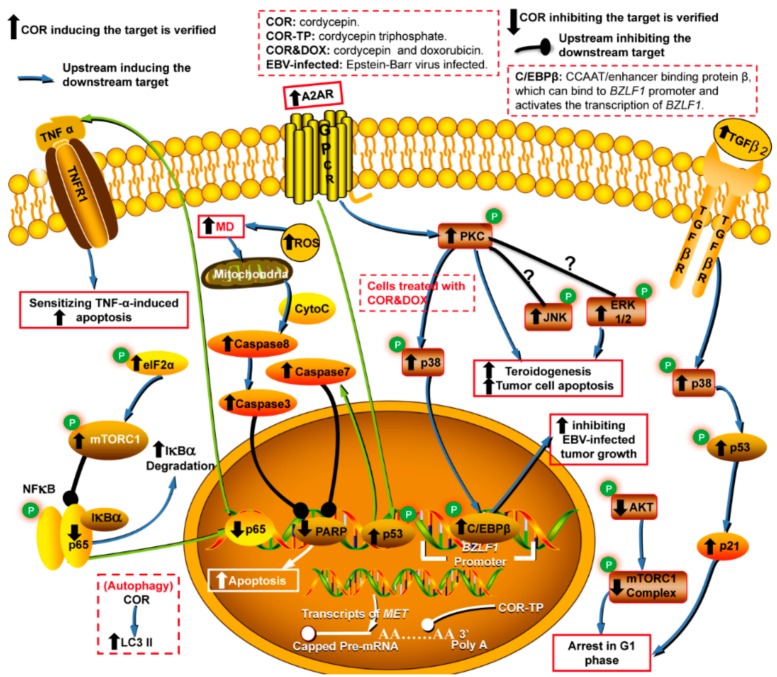
Cellular targets of tumor affected by cordycepin via various signal pathways. Note: ROS: reactive oxygen species. C/EBPβ: CCAAT/enhancer binding protein β, which can bind to *BZLF1* promoter and activate the transcription of *BZLF1* [17]. MD: mitochondrial disfunction. These abbreviations are also used in this paper.

**Figure 3:**
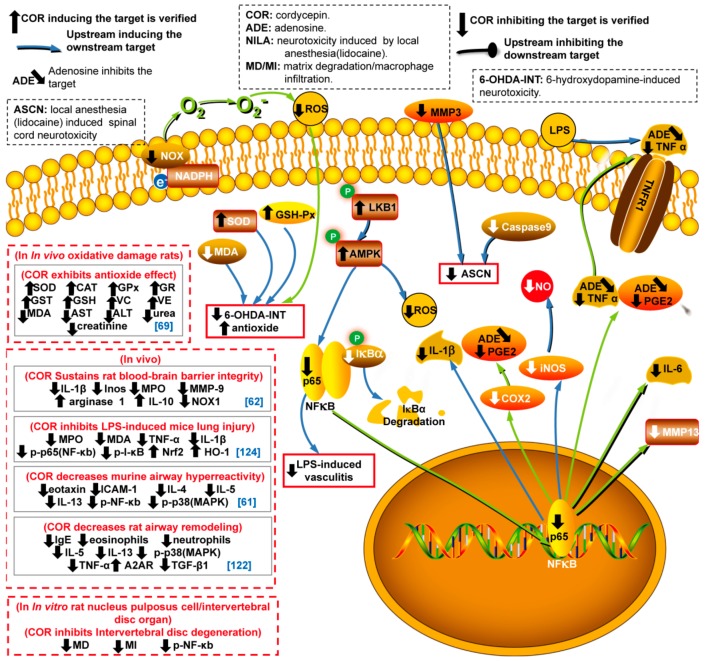
Anti-inflammatory and anti-oxidant targets affected by cordycepin via various signal pathways. Note: SOD: superoxide dismutase. GSH-Px: glutathione peroxidase. MDA: malondialdehyde. 6-OHDA-INT:6-hydroxydopamine-induced neurotoxicity. VC: Vitamin C. VE: Vitamin E. IL-1β: interleukin-1 beta. iNOS: inducible nitric oxide synthase. PGE2: prostaglandin E2. NO: nitric oxide. COX-2: cyclo-oxygenase. NF-κB: nuclear factor kappa-B. iNOS: inducible nitric oxide synthase. IgE: immunoglobulin E. ICAM-1: intercellular cell adhesion molecule-1. HO-1: heme oxygenase-1. These abbreviations are also used in this paper.

**Figure 4:**
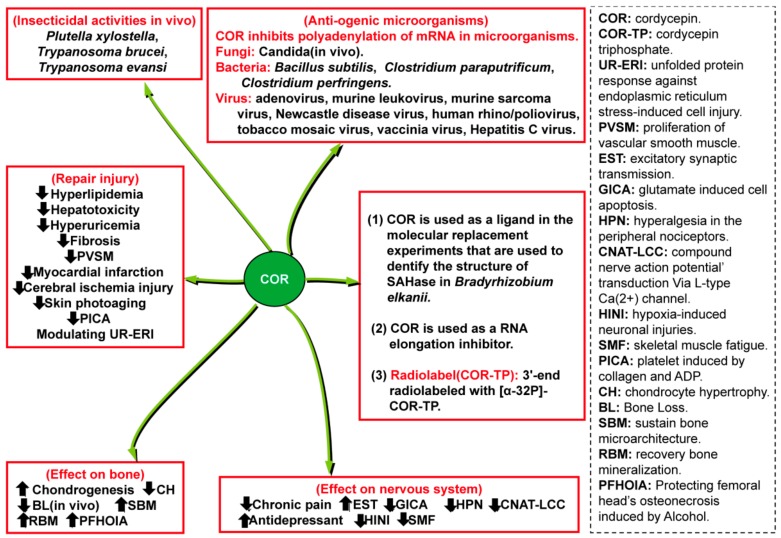
Other medicinal value and biological applications. Note: COR can also inhibit excitatory synaptic transmission [18] and have neuroprotective effects [19]. ER-COR has antiplatelet effects [20]. The [α-32P]-COR-TP is used for 3′-end radiolabeled RNA fragments [21].

Cancer, a major threat to public health, is an important cause of death. Recently, the World Health Organization reported that 18.1 million new cases of cancer and 9.6 million cancer deaths occurred in 2018 [1]. In China, cancer has been the leading cause of death since 2010 [2]. Notably, cordycepin (3′-deoxyadenosine [3], COR), discovered in the broth of *Cordyceps militaris* [4], has received a large amount of attention due to its enormous therapeutic potential, as well as its effects on intracellular signal transduction and cell adhesion [5]. COR interferes with many pathological processes via the inhibition of mRNA polyadenylation [6,7] and the regulation of a variety of targets involved in various cellular processes, such as anticancer(apoptosis and autophagy induction, leukemic stem cell (LSC) elimination, cell cycle arrest, and antimetastatic, anti-invasion, and antiproliferation activities), anti-inflammatory, antioxidant, antipathogenic, insecticidal, antihyperlipidemic, antihepatotoxic, antifibrotic and neuroprotective activities, skin photoaging protection, skeletal muscle fatigue repression, protection from ischemia/reperfusion-induced injury and bone protection (Figure 1, Figure 2, Figure 3 and Figure 4). Several potential reasons may account for the multiple bioactivities of COR: (i) Cordycepin triphosphate (COR-TP) may be initially generated through the phosphorylation of COR [8], which is incorrectly recognized as ATP binding to targeted enzymes and replaces ATP due to the highly structural similarity between COR-TP and ATP. Enzymes also erroneously recognize COR as adenosine, which causes abnormal purine metabolism. (ii) COR and/or COR-TP inhibit targets by replacing ATP binding to targeted protein kinases with the highly structural similar COR-TP [9] and/or the activation of protein phosphatases [10]. (iii) COR and/or COR-TP directly activate protein kinases, such as AMPK [11] potentially because of an increase in the AMP/ATP ratio. (iv) COR and/or COR-TP interrupt mRNA polyadenylation because of the erroneous recognition of COR-TP as ATP by PolyA polymerase [7]. In addition, COR has the potential to act as a ligand [12] and a RNA elongation inhibitor [13], and it can specifically inhibit transcription efficiency [14].

However, the large-scale production of COR is a significant challenge. Hence, COR serves as an important marker for the quality control of *C. militaris*. The COR content of *C. militaris* is significantly higher than that of other currently known fungi. The maximum production of COR obtained in liquid-cultured *C. militaris* by mutant G81-3 and in the fruiting body of wild *C. militaris* reached 14.3 g/L [22] and 9.45 mg/g [23], respectively. For the same *C. militaris*, the COR content in the fruiting body was significantly higher than that in the mycelia [24]. Notably, the COR biosynthetic mechanism in *C. militaris* was completely elucidated [24] through a comparative analysis of the conserved genes of two phylogenetically distant and COR-producing species, *Aspergillus nidulans* [25] and *C. militaris*. Intriguingly, pentostatin (PTN), which protects COR from deamination by adenosine deaminase (ADA), was first discovered in the fruiting body of *C. militaris* (FB-CM) rather than in the mycelia [24]. 

Although the medicinal potential of COR [26,27,28], the COR analogs in mushrooms [29] and the pharmacological effect of COR on male reproduction [30] have been extensively reviewed, little attention has been paid to the medicinal targets, cytotoxicity, delivery carriers, stability, and biological applications of COR, the metabolic mechanisms in COR-producing fungi, and the pros/cons of COR in clinical applications, and the clinical potential of the external use of COR. In fact, these aspects are thought to be significant issues for the medicinal applications of COR. In addition, the mechanisms of disease treatments with chemotherapeutic agents, such as COR, are sophisticated. It is essential to improve the efficiency of chemotherapeutics agents, to design analogs suitable for clinical treatment, and to develop novel therapeutic strategies via in-depth understanding of COR. Therefore, this review briefly summarizes these aspects. In addition, we discuss new approaches that will greatly contribute to uncovering the metabolic mechanisms of all facets of COR in *C. militaris*.

## 2. Anticancer Activity in Similar Signaling Pathways

### 2.1. Induction of Apoptosis and Promotion of Autophagy

Apoptosis, a type of programmed cell death (type I cell death), is characterized by marked changes inmorphology and other biochemical markers, that involves the activation of caspases via extrinsic (cell membrane receptors), intrinsic mitochondrial-related and endoplasmic reticulum (ER) stress-related pathways. COR suppresses the growth of cancer cells by blocking formation of the polyA tail on mRNA at the transcriptional level and affects various targets in different signaling pathways [7] (Figure 1 and Table S1). Studies have shown that COR induces the apoptosis of cancer cells via cell membrane receptors [31], mitochondria [32] and PI3K/Akt [33] signal pathways. Mechanistically, COR not only activates death receptor 3(DR3) [34], adenosine G protein-coupled receptors (GPCRs) [31], tumor suppressor p53 [35] and pro-apoptotic proteins (Bax and caspases) [32] but also inhibits anti-apoptotic proteins (BCL-2 and BCL-xL) [36] and PI3K/Akt [33]. 

COR induces the apoptosis of cancer cells via extrinsic pathways, including the activation of DR3/caspase-8/caspase-1 pathway in the in vitro colonic cancer cell line HT-29 [34] and the stimulation of adenosine A1 receptor (A1R)/adenosine A3 receptor (A3R)/intracellular calcium (IC)/calpain/caspase7/poly adenosine-diphosphate-ribose polymerase (PARP) pathway in the in vitro thyroid cancer cell line CGTH W-2 [31], and by increasing the level of tumor necrosis factor-α (TNF-α) bound to tumor necrosis factor receptor (TNFR) in hepatocellular cancermice in vivo [37]. Notably, COR stimulates A1R and A3R to a greater extent than it stimulates the adenosine A2A receptor (A_2A_R) and adenosine A2B receptor (A_2B_R) [31].

In addition, COR stimulates apoptosis of cancer cells through intrinsic pathways, such as mitochondrial, PI3K/Akt and autophagy pathways. For the mitochondrial pathway, COR not only affects the proapoptotic/anti-apoptotic proteins in mitochondria but also causes mitochondrial dysfunction. Previous work reported that COR induces the apoptosis of breast cancer cells in vitro via the Bax/cytochrome c(cyt c)/caspases-9/caspases-3/PARP pathway in the MDA-MB-231 cell line [32]. Notably, COR stimulates mitochondrial dysfunction by modulating the intracellular level of gaseous signaling molecules, such as increasing reactive oxygen species (ROS) and decreasing nitric oxide(NO). Mitochondrial dysfunction induces the release of Bax and Cyt c from the mitochondria to the cytoplasm and activates a downstream pathway that includes caspases. Previous studies demonstrated that COR induces the apoptosis of cancer cells by enhancing intracellular ROS via the ROS/caspase-8/caspase-9/caspase-3/caspase-5/caspase-7 pathway in human leukemia cell lines (U937 and THP-1) in vitro [38] and via an antioxidant protein-mediated pathway (GPX/superoxide dismutase (SOD)/Catalase/ROS/Bax/BCL-2/caspase-9/caspase-3) in brain cancer cell lines (SH-SY5Y and U-251) in vitro [36]. Nevertheless, COR decreases ROS levels in the mononuclear cells of peripheral blood samples from both healthy subjects and subjects with Kawasaki disease [39]. This finding suggests that COR exhibits contrasting effects under different conditions. In addition, COR induced apoptosis in vitro in the human lung cancer cell line A549 by inhibiting the intracellular NO via the nitric oxide synthase (NOS)/NO/EKR/GSK-3β/Slug/Bax /caspase-3/PARP pathway [40]. Furthermore, COR stimulates apoptosis by inhibiting the PI3K/Akt pathway (PI3K/Akt/hTERT/telomerase) inleukemia cell line (U937 and THP-1) in vitro [33] and induces autophagy (type II cell death) in the breast cancer cell line MCF-7 in vitro [32].

### 2.2. Cell Cycle Arrest

The cell cycle is regulated by cyclins, cyclin-dependent kinases (CDKs) and cyclin-dependent kinase inhibitors (CDKIs). The complex of a cyclin and the corresponding CDK, which is inhibited by CDKIs, arrests the cell cycle. Previous research demonstrated that COR promotes cancer cell cycle arrest at certain cell cycle phases by inhibiting cyclins (cyclins D1 [41,42], E [42] and B1 [43]), repressing CDKs(cdc2) [43] and inducing CDKIs (p21 and p27) [42] (Figure 1 and Table S1). Additionally, previous work demonstrated that COR arrests the cancer cell cycle at the G1 phase in vitro via the PI3K/Akt pathway (Akt/GSK-3b/β-catenin/cyclinD1) in the leukemia cell line U937 [41] and the CDKIs (p21/p27)/Cyclin(D1 and E) pathway in the leukemia cell line BCRC60176 [42]. In addition, COR promotes cancer cycle arrest at the G2/M phase via the checkpoint kinase 1 (CHK1)/cylinB1-cdc2 complex pathway in the cervical cancer cell line (HeLa) in vitro [43]. Notably, COR induces cell cycle arrest at the G0/G1 phase in in vitro lung cancer cell line H1975 via decreasing the level of phosphorylated epidermal growth factor receptor (EGFR), subsequently causing a decrease in phosphorylated AKT and phosphorylated ERK1/2. COR binds to the tyrosine kinase domain of EGFR and interferes with ATP binding to prevent EGFR phosphorylation [9]. Therefore, COR and its derivatives, as novel EGFR inhibitors, have the potential for further medicinal applications.

### 2.3. Suppression of Metastasis, Invasion, Proliferation and Mesenchymal Stem Cells

Cancer, a type of malignant tumor, is formed from benign tumors through a series of processes that involve embryonic cells, mesenchymal stem cells, fibroblasts, fibrocytes and malignant tumor cells. In contrast to benign tumors, cancer is characterized by rapid progression, including metastasis, invasion and proliferation. The malignant properties of tumors are not only controlled by cancer cells but also driven by microenvironment of the tumor cells [44], including a variety of nonmalignant cells (macrophages, fibroblasts, inflammatory cells and mesenchymal stem cells) [44], the paracrine signaling exchange of cytokines [45], cell adhesion factors (β-catenin and N-cadherin) [46], growth factors [45], tumor cell release factors [47] and transforming growth factor-beta (TGF-β) [48].

As shown in Figure 1 and Table S1, Previous studies demonstrated that COR represses the metastasis of glioblastoma cancer cells via the inhibition of the integrin β1 receptor pathway (integrin β1 receptor/FAK/paxillin/Akt) by lysosomal degradation in in vitro cell lines (ANM, U87MG and LN229) in vitro [10]. Noteworthily, COR can activate protein phosphatases to inhibit targets (integrin β1 receptor, FAK, paxillin and Akt) [10]. In addition, COR inhibits migration and invasion via MMP-mediated pathways in vitro, for example, COR suppressed the AKT pathway (AKT/MMP-9/MMP-2) in the prostate cancer cell line LNCaP [49], repression of MMP-9/MMP-2 in in vitro lung cancer cell line CL1-0 [50] and inhibited the AP-1 pathway (TPA/AP-1/MMP-9) in the breast cancer cell line MCF-7 [51]. In addition, COR has been demonstrated to repress epithelial-mesenchymal transition via the upregulation of E-cadherin and downregulation of N-cadherinin in vivo oral cancer-bearing mice [52].

In addition, as shown in Figure 1 and Table S1, COR inhibits proliferation by activating the adenosine 5′-mono-phosphate-activated protein kinase (AMPK) pathway (AMPK/mTORC1/MDR /HIF-1α) in vitro in the gallbladder cancer cell line GBC-SD [11], by inducing the p38 MAPK pathway (p38 MAPK/ERK) in vitro in the renal cancer cell line 786-O [53], and by inactivating tyrosinases in vitro in the lung cancer cell lines (A549 and Caalu-3) [54]. Notably, AMPK is generally activated in response to a high adenosine 5′-monophosphate AMP/ATP ratio [55]. It is speculated that COR may cause an increase in the AMP/ATP ratio, which induces AMPK. The speculation requires further investigation. Moreover, COR inhibits the progression of leukemia by inhibiting leukemic stem cells (LSCs) and suppressing leukemia-stromal interactions (LSIs). Both LSCs and LSIs can develop resistance to chemotherapeutics. Mechanistically, COR induces apoptosis of leukemia cells via the autocrine signaling pathway in in vitro cell lines (U937/K562) by decreasing cell adhesion factors (β-catenin and N-cadherin) and via the paracrine signaling pathway in vivo in the U937/K562-inoculated mice by inducing Dickkopf-1 (DKK1, a wnt/β-catenin inhibitor [56]), and restraining the NFκB pathway [46]. The anticancer properties of COR in other in vivo studies are shown in Figure 1/Table S1 and are labelled in the square brackets using the blue numbers associated with the corresponding references.

## 3. Antitumor Activity

### 3.1. Apoptosis Inhibition, mTOR Repression and Cell Cycle Arrest

The inhibition effect of COR on benign tumor cells is less pronounced than that on highly aggressive cancer cells [57]. COR does not impact healthy and noncancerous cells [54]. COR induces apoptosis of tumor cells through mitochondria (caspases) and the mTOR and autophagy pathways, as well as by inhibiting ER stress-induced injury (Figure 2 and Table S2). COR promotes the apoptosis of tumor cells through various pathways in vitro, for example, COR represses the transcription of *MET* in the multiple myeloma cell line MM.1S [58], activates the ROS-mediated mitochondria pathway (ROS/caspase-8/caspase-3/PARP) and induces autophagy in the Leydig tumor cell line MA-10 in a dose-dependent manner [59] and stimulates p53 signaling (A_2A_R/p53/caspase-7/PARP) in the glioma cell line C6 (malignant tumor cell) [60]. In addition, COR can bidirectionally regulate mTOR signaling under different conditions. The literature indicates that COR represses tumor growth in vitro by inhibiting mTOR pathway (AKT/mTOR) in the Leydig tumor cell line MA-10 [59]; however, in the healthy renal cell line NRK-52E and in other cell types, COR induces the mTORC1 pathway (elF2α/mTORC1/p65 of NF-κB), which then sensitizes cells to TNF-α-induced apoptosis [61]. Similar to previous work [61], COR was found to suppress ER stress-induced apoptosis in vitro in the NRK-52E cell line [62]. In other words, COR kills TNF-α-treated NRK-52E cells and has a protective effect on NRK-52E cells against ER stress-induced apoptosis, suggesting that COR bidirectionally regulates apoptosis. In addition, COR promotes the cell arrest of tumor cells at the G2/M phase in vitro by inducing TGFβ2-mediated extrinsic pathway (TGFβ2/p38/p53/p21) in the Leydig tumor cell line MA-10 [59].

### 3.2. Suppression of Tumor Growth via the GPCR/PKC Signaling Pathway

MAPK cascades are important stress-responsive signaling pathways that govern a wide variety of functions implicated in tumor growth. MAPK signaling pathways consist of three components: MAPKs, MAPK kinases and MAPK kinase kinases. GPCRs, including adenosine receptors, can activate the MAPK pathway [63]. Phospholipase C (PLC) is induced by GPCR and stimulates protein kinase C (PKC) to activate the downstream event. COR inhibits tumor cell growth through MAPK pathways (Figure 2 and Table S2). COR significantly promotes steroidogenesis via the PKC/JNK/ERK1/2 pathway rather than via the PKA/PI3K/p38 MAPK pathway in vitro in the Leydig tumor cell line MA-10 [64]. In addition, COR inhibits tumor growth via the PKC/p38 MAPK/transcription factor (C/EBPβ) pathway in vivo in Epstein-Barr virus-infected mice [17]. 

## 4. Anti-Inflammatory and Anti-Oxidant Activities in Different Cell Lines

### 4.1. Anti-Inflammatory Activity

COR has been demonstrated to exhibit anti-inflammatory activity in different cell lines [39,65,66]. Mechanistically, this effect is ascribed to the protection of IκB-α from degradation, which inhibits NF-κB, and to the inactivation of MAPK [67]. The effect of COR on NF-κB, AMPK and MAPK directly or indirectly cause the downregulation of proinflammatory and inflammatory factors (PGE2 [65,66], COX-2 [65,66], Inos [66], IL-1β [65,66], and TNF-α [65]) and the upregulation of anti-inflammatory factors (interleukin proteins) [68] (Figure 3 and Table S3). COR exhibits anti-inflammatory properties via various signaling pathways in vitro in different cell lines. In RAW 264.7 cell lines, COR inhibits inflammation in vitro via the activation of the LKB1 pathway (LKB1/AMPK/NF-κB and ROS) during the treatment of Kawasaki disease [39] and represses lipopolysaccharide (LPS)-induced inflammation by inhibiting TNF-α and PGE2 [65]. In IL-1β-induced human osteoarthritis chondrocytes in vitro, COR exhibits anti-inflammatory effect via the inhibition of the p65 NF-κB pathway (IκB-α/p65 NF-κB/COX-2/PGE2/iNOS/NO/MMP-13 /IL-6) [66]. Moreover, the anti-inflammatory properties of COR have been observed in the HMC-1 cell line, where COR inhibits inflammatory factors (IL-13/IL-6/TNF-α/IL-1β) in atopic dermatitis treatment [69], and in vitro in rat spinal cord dorsal root ganglia neurons, where COR inhibits caspase-9 and MMP-9 in local anesthesia-induced spinal cord neurotoxicity [70].

### 4.2. Anti-Oxidant Activity

Endogenous enzymes that scavenge free radical and act as antioxidants become dysfunctional with age, which causes the aberrant accumulation of ROS. Previous work demonstrated that the anti-oxidant activity of COR and adenosine from water extracts of C. militaris is limited [71,72]. Mechanistically, as shown in Figure 3 and Table S3, COR exhibits anti-oxidant activity via a decrease in intracellular ROS by regulating antioxidant and oxidant enzymes and antioxidants, including decreasing the level of NADPH oxidase in vitro in the tubulointerstitial fibrosiscell line HK2 [73], decreasing the level of malondialdehyde (MDA) and activating SOD and glutathione peroxidase (GSH-Px) to protect cell line PC12 against 6-hydroxydopamine-induced neurotoxicity in in vitro studies of Parkinson’s disease [74]. In addition, COR decreases ROS in vivo in radical-induced oxidative damaged rats [75].

The anti-inflammatory and anti-oxidant properties of COR in other in vitro and in vivo studies are shown in Figure 3 and Table S3.

## 5. Other Medicinal Values and Biological Applications

### 5.1. Insecticidal Activity and Inhibition of the Growth of Pathogenic Microorganisms

COR, which is very similar to adenosine but lacks a 3′-hydroxyl group, can be erroneously identified and replaces nucleosides and interrupts the polyadenylation of mRNA, causing dysfunction and inhibiting the growth of pests and pathogenic microorganisms (Figure 4 and Table S4). Previous work demonstrated that COR inhibited the growth of pathogenic microorganisms, including *Bacillus subtilis* [76], adenovirus [77,78], murine leukovirus [79], murine sarcoma virus [80], Newcastle disease virus [81], human poliovirus [82], tobacco mosaic virus [83], vaccinia virus [84], hepatitis C virus [85], *Clostridium paraputrificum* and *Clostridium perfringens* [86] and *Candida* [87]. The effect of COR on human immunodeficiency virus (HIV) is not well understood, and additional investigations are needed to tackle this issue. Notably, the efficacy of a mosaic adenovirus serotype 26-based HIV-1 vaccine that exhibits excellent immune responses, safety and tolerability in humans and rhesus monkeys are being evaluated in phase 2b clinical trials in sub-Saharan Africa [88].

In addition, COR exhibits insecticidal effects (Figure 4 and Table S5). COR can induce cell death in pests, including *Plutella xylostella* [89], *Trypanosoma brucei* [90] and *Trypanosoma evansi* [91]. The combination of COR (2 mg/kg) and PTN (0.2 mg/kg) has significant therapeutic potency and decreases toxicity in *T. evansi*-infected mice [92]. Notably, COR induces programmed cell death rather than repression of chitin synthesis in vivo in larvicidal *P. xylostella* [89]. In addition, COR exhibits antifungal activity, such as against different *Candida* isolates [87]. Considering that chitin is the major component of the fungal cell wall, a combination of COR and chitin synthase inhibitors is a promising strategy in insecticidal and antifungal applications.

### 5.2. Inhibition of External Factor-Induced Injury

External factors often result in a series of secondary pathological effects and/or alter the function of healthy tissues and organs, causing longterm disability or death. Studies have revealed that COR enhances injury repair via several pathways [93,94].

Skin aging is a degenerative physiological process induced by both internal and external factors [93]. Previous work demonstrated that COR suppresses skin photoaging [95], inhibits fibrosis and exhibits antioxidant properties [94] (Figure 4 and Table S5). In previous skin photoaging studies, COR represses skin photoaging in vitro by blocking UVB-induced NF-κB activation, and the NF-κB inactivation subsequently downregulates *MMP-1* and *MMP-3* expressions. The inhibition of MMP is a promising strategy in skin cancer and photoaging therapy [95]. Moreover, COR represses lung fibrosis by increasing E-cadherin expression and decreasing vimentin levels [96] and inhibits kidney fibrosis by inactivating targets (CAGA box, BRE and Smad1/2/3) and stimulating eIF2α [97]. In addition, COR plays a significant role in suppressing atopic dermatitis [69]. Therefore, COR has cosmeceutical potential with additional medicinal applications. 

Trauma induces many physiological changes that subsequently cause complications, such as ischemia injury. As shown in Figure 4 and Table S5, previous work indicated that COR inhibits ischemic injury in vivo in myocardial infarction [98] and cerebral ischemia injury [99]. Notably, remote ischemic preconditioning can also protect target tissues/organs from injury and has notable developmental potential because of its clinical safety, simplicity, and acceptance. Pre-treating target tissues/organs with COR coupled with remote ischemic preconditioning is considered to be an efficient and promising strategy to overcome ischemic injury. In addition, trauma may also cause physiological complications, including pathogenic microorganism infection, physiological changes in local blood vessels, and provisional or chronic pain. COR can markedly inhibit growth of specific pathogenic microorganisms, restrain these physiological changes and repress neuropathic pain due to its multiple bioactivities. COR suppresses the proliferation of vascular smooth muscle exclusively [100]. In addition, COR can markedly suppress hyperactivity of nerve tissue by interrupting the L-type Ca^2+^ channel-mediated transduction of compound nerve action [101], blunting the peripheral nociceptors [102] and significantly suppress chronic pain by decreasing the level of PEG2 [103]. In addition, COR markedly decreases skeletal muscle fatigue-induced muscle strain [104]. These results suggest that COR has the potential to be used as an external drug for trauma and muscle strain as well as for remote ischemic preconditioning.

### 5.3. Inhibition of Internal Factor-Induced Injury

Compared with external factors, internal factors, which include ER stress, over-adipogenesis in tissues/organs, alcohol-induced dysfunction of tissues/organs and neurological disorders, may cause several serious pathological changes, such as ER stress-induced injury, hepatotoxicity and neurodegeneration. As shown in Figure 4 and Table S5, COR protects against ER stress-induced injury by inducing eIF2α and inhibiting GADD34 [62]. In addition, COR suppresses adipogenesis-induced and alcohol-induced hepatotoxicity [105,106], inhibits hyperlipidemia by activating AMPK [107,108] and inhibits depression by promoting *GluR1* expression [109]. 

### 5.4. Promotion of Chondrogenesis and Inhibition of Bone Loss

Currently, osteoporosis represents a marked public health challenge. Previous work revealed that COR protects against osteoporosis as a result of increased bone generation and inhibited bone loss (Figure 4 and Table S5). Mechanistically, COR alleviates osteonecrosis via a decrease in ROS [110], protects the femoral head from alcohol-induced injury by activating β-catenin and Runx2 [111] and induces chondrogenesis through the stimulation of PI3K and MMP-13 [112]. 

### 5.5. Biological Applications of COR and COR-TP

COR structurally resembles adenosine, except that COR lacks a 3′ hydroxyl group, which significantly enhances the ability of COR to form transition metal complexes in the form of di-, tri- and tetra-dentate ligands [12]. COR and COR-TP have many biological applications (Figure 4 and Table S5). COR has been used as a ligand in molecular replacement experiments that are used to identify the molecular structure of SAHase in *Bradyrhizobium elkanii* [113]. Furthermore, since COR inhibits the PolyA formation of mRNA, the presence of COR can cause premature transcription termination. Previous research used COR as an RNA elongation inhibitor, and bromine in BrUTP was used to elucidate the structure of active rRNA gene in the nucleolus. Pretreated with COR, premature rRNA elongation in isolated nucleoli incubated with BrUTP was terminated. The release of inhibition enabled the immediate elongation, and the starting sites of BrUTP incorporation were identified by immunogold labelling detection method [13]. Therefore, COR efficiently inhibits premature rRNA elongation and can be used to control the transcriptional reaction. In addition, COR-TP, which exhibits a high level of structural similarity to ATP, may be derived from COR before directly blocking RNA synthesis in mutant *Saccharomyces cerevisiae* [8], and COR interferes with the efficiency of mRNA polyadenylation rather than terminating mRNA elongation [6]. Mechanistically, the structure of human poly(A) polymeraseγ, which catalyzes the polyadenylation of mRNA, is identified using COR-TP (chain terminator) and Ca^2+^ (divalent cation) which bind to the active site of poly(A) polymeraseγ [114]. The binding of COR-TP and Ca^2+^ to poly(A) polymeraseγ may contribute to illuminating the reasons of COR-induced eryptosis in a Ca^2+^-dependent manner [115] and the COR-TP-mediated interruption of polyadenylation [58]. Hence, COR and COR-TP are considered specific inhibitors of RNA synthesis in various applications, such as the use of COR to investigate of the amounts of rhythmic RNAs [116].

## 6. Pros and Cons of COR in Clinical Applications

### 6.1. Pros of COR in Clinical Applications

COR exerts curative effects, including significant(+++), less pronounced(++), slight(+) and no curative potency(-), on different types of diseases by affecting many molecular targets involved in various cellular signaling processes. A number of the advantages of COR in clinical applications are shown in Table 1.

### 6.2. Cons of COR in Clinical Applications

(a) Possible toxicity to healthy cells: COR is toxic to malignant cancer cells [57] and nontoxic to healthy cells [54,134]. However, previous have also stated that COR exhibits toxicity towards healthy erythrocytes [115] and impairs healthy organs (liver, and kidney) in vivo in mice [91].
(b) Unfavorable pharmacokinetics: Since COR quickly loses its activity due to in vivo ADA and stomach acid conditions [24], COR has a short half-life and is rapidly eliminated [135]. The resistance of ADA to COR represents a significant issue because ADA can deaminize the adenosine analog COR.
(c) Low solubility in water: The solubility of COR plays a vital role in drug storage and efficient therapeutic efficiency. The low solubility of COR in water causes low chemical stability, poor oral bioavailability and low cellular uptake. Previous work demonstrated that phosphate-buffered saline (PBS, pH 4.0) is a suitable solvent for COR in intravenous and oral treatments at low doses. Propylene glycol (PPG) is more applicable than PBS at pH 4.0 as a COR solvent for oral treatments [136].
(d) Complex mechanisms of action: COR inhibits and/or induces multiple medicinal targets in a dose-dependent, condition-dependent and nonspecific manner: Different concentrations of COR exhibit diverse effects on MA-10 mouse Leydig tumor cells (MLTCs). A low concentration of COR activates the caspase-3/caspase-6/caspase-7/caspase-8/PARP pathway in MMLTCs, while a high dose of COR markedly increases the levels of p-AKT and p-mTOR and stimulates only caspase-3 rather than caspase-6/caspase-7/caspase-8 [59]. In addition, since COR is less reactive to PARP than to a specific inhibitor of PARP, COR is considered to exhibit PARP-inhibitory activity rather than specifically inhibit PARP [57]. In addition, COR exerts bidirectional regulatory activity under disparate stress-induced conditions [61,62] and in different cell types [59,61].
(e) Drug resistance: Multidrug resistance, one of the major obstacles, markedly decreases the curative potency of anticancer agents and the treatment of other diseases.
(f) Clinical safety and potency: The safety and efficacy of COR as a TdT-positive leukemia treatment is currently being evaluated in phase Ⅱ clinical trials [137]. Currently, the clinical application of COR in the treatment of TdT-positive leukemia is not permitted.

### 6.3. Medicinal Strategies for Promoting COR Efficiency and Safety

(a) Inhibition of deamination by ADA. Three strategies have been used to approach this problem. (i) combined use of COR and ADA inhibitor; (ii) natural and designed ADA-resistant derivatives of COR; and (iii) nanocarrier for ADA-resistance. In strategy i, the combination of COR and an ADA inhibitor markedly improves the stability of COR. Efficient ADA inhibitors, such as actinomycin D [116], erythro-9-(2-hydroxy-3-nonyl)-adenine [135] and PTN [24], significantly enhance the bioavailability of COR. In strategy ii, multiple natural derivatives of COR in mushrooms [29] and their therapeutic value [138] have been extensively reviewed. In addition, designed ADA-resistant derivatives of COR can overcome treatment failure, such as the high bioavailability pro-cordycepin(*N*-acyloctanoylcordycepin), which is 4(time of maximum concentration)/30(maximum concentration)/68(area under concentration) times higher than that of COR [139], and ADA-resistant 2-fluoro-3′-deoxyadenosine has similar cytotoxicity (IC_50_) values of 2.44 ± 0.69 μM (this compound alone) and 2.13 ± 0.87 μM (this compound co-incubated with ADA inhibitor PTN) while COR has markedly different IC_50_ values of 0.10 ± 0.03 μM(COR co-existing with PTN) and over 100 μM (COR alone) on MOLT4 cells in vitro [140]. Interestingly, an efficient and eco-friendly biotransformation system for generating 5′-O-acetylcordycepin at the 25-g scale and a 96.2% isolated yield in solvent 2-methyltetrahydrofuran was developed, and Novozym 435 (an immobilized *Candida antarctica* lipase B) used in this system retains 63% of its original activity after 7 recycling batches [141]. In strategy iii, a nanocarrier composed of layered double hydroxides was developed to prevent COR from deamination by ADA, such as [Mg–Al–cordycepin] nanohybrids. At the same concentration, this nanohybrid inhibit the growth of U937 cells at a rate that is 3.185 times higher than that of COR [142].
(b) Bypassing gastric acid conditions. The bioactivity of COR quickly decreases under gastric acid conditions. Hence, COR carriers, including gelatin type A nanoparticles [134] and transferrin-conjugated liposomes [143] have been developed to approach this problem.
(c) Decreasein toxicity. Several potential strategies are available to decrease the toxicity of COR: (i) low dose of treatment; (ii) natural and designed derivates; (iii) COR carriers for region-targeted treatment and the specific accumulation of COR. In strategy i, an appropriate dose of COR should be selected. In strategy ii, few studies have discovered and designed novel derivatives of COR, such as designed *N*-octanoylcordycepin which exhibits lower metabolic velocity and higher bioavailability than COR [139]. In strategy iii, appropriate region-targeted drug carriers, such as transferrin-conjugated liposomes for COR delivery to liver cancer cells [143] and gelatin type A nanoparticle for COR delivery to lung cancer cells [134], increase the specific accumulation of COR at the desired region, decrease the total dose of COR and reduce unintended extravasation into healthy regions.
(d) Overcoming drug resistance. Drug resistance presents a serious challenge when diseased cells develop resistance to COR over time through various mechanisms that markedly reduce the curative potency of COR. Previous work uncovered several mechanisms of novel drug resistance, such as extracellular vesicles that mediate drug resistance due to direct exportation [144], cytotoxic drugs sequestration [145], and decreased effective concentration of the drug. COR resistance can be promoted through extracellular vesicle-mediated pathways. In addition, the combination of COR and other agents can efficiently overcome drug resistance. Water or ethanol extract rich in COR of fruiting bodies and/or mycelium of *C. militaris* (ER-COR) is also a potential strategy to conquer drug resistance.
(e) The low cost and high potency of ER-COR. The proliferation of renal carcinoma cells is more efficiently suppressed by the ER-COR from FB-CM than the ER-COR from mycelia [119]; moreover, ER-COR stimulated apoptosis more effectively than COR alone in vitro in human leukemia cells [53]. These effects may also be due to other components of FB-CM, such as PTN [24] and adenosine. In addition, ER-COR can strengthen immunity by increasing the level of cytokines (IL-2/IFN-γ/TNF-α) in vitro in splenocytes and cytokines (IL-2/IFN-γ/TNF-α/IL-10) in vivo in immunosuppressed mice [146]. Nevertheless, the bioactive components of ER-COR are distinct due to their recognition of different targets. In vitro studies of human hepatocellular carcinoma cells revealed that the inhibitory effects of COR on ERp57 are more efficient than those of zhankuic acid A and adenosine, while the inhibition of PGK-1 mediated by COR is less pronounced than that mediated by zhankuic acid A and adenosine [124].

## 7. Metabolic Mechanisms of COR in COR-Producing Fungi

### 7.1. Scientific Name of the Caterpillar Fungus Called DongChongXiaCao in Chinese

The teleomorph and anamorph of DongChongXiaCao have been scientifically named according to the rule “One Fungus=One Name” [147]. Although the teleomorph of DongChongXiaCao was previously named *Sphaeria sinensis* Berk. [148] and *Cordyceps sinensis* (Berk.) Sacc. [149,150], *Ophiocordyceps sinensis* is the modern taxonomic name for DongChongXiaCao [151]. This fungus is found only on the Qinghai-Tibet Plateau of China [152]. Although approximately twenty names have been used in the past [153], *Hirsutella sinensis* is the scientific name of the anamorph of DongChongXiaCao [154]. 

### 7.2. COR-Producing Fungi

*Cordyceps* and *Ophiocordyceps* are the primary COR-producing fungi (Table 2). The *C. militaris* fungus produces a higher level of COR than other fungi. Mutant G81-3 [22] and wild CICC 14014 [155] strains of *C. militaris* produced a maximum of 14,300 and 7350 μg/mL COR, which would be efficient for industrial applications. Notably, the total amino acid content of the mycelia of *O. sinensis*, which does not contain detectable levels of COR, exceeds that of the FB-CM. The COR content in the FB-CM reached 1.743 mg/g, while no COR [156] or trace amounts of COR [157] were detected in the fruiting body of *O. sinensis*. In addition, the fruiting bodies of both *C. sinensis* and *Ophiocordyceps xuefengensis*, which are very similar [158], produce trace amounts of COR [159,160]. Intriguingly, previous work has emphasized that massive amounts of COR is produced during the development of FB-CM but not during the development of the mycelia [24]. Therefore, a comparative analysis of similar groups (*Cordyceps* vs *Ophiocordyceps*) or the same fungus at different development phases would contribute to the elucidation of the key factors involved in COR metabolic pathways(biosynthesis and cellular detoxification).

### 7.3. COR Metabolic Mechanisms in C. militaris under Hypoxia, Light and Heat Stress

The mycelia of *C. militaris* obtained under liquid surface culture conditions produced a higher level of COR than the mycelia obtained under a submerged culture condition [22]. Mechanistically, cytochrome P450 oxidoreductases, including heme, are markedly enriched in the static liquid surface culture, which indicate a hypoxic condition. Hypoxic conditions significantly increase the levels of hypoxia-associated proteins, adenylosuccinate synthase and phosphoribosyl-aminoimidazolesuccino-carboxamide synthase involved in purine nucleotide metabolism [170]. Similar results that hypoxia stress significantly promoted gene expansion of cytochrome P450 were observed during the fruiting of *O. sinensis* [176]. Since COR can modulate intracellular ROS levels in diseased and healthy cells, COR biosynthesis may be associated with intracellular ROS modulation in *C. militaris*. The detailed mechanism requires further investigation. 

Certain types of light can induce COR biosynthesis via photoacceptor-mediated signal pathways in *C. militaris*. Studies have demonstrated that the regulation of COR biosynthesis in *C. militaris* is associated with blue-light receptors, including Cmwc-1(wc-1 in *C. militaris*) and CmCRY-DASH (CRY-DASH in *C. militaris*). Cmwc-1 upregulates adenylosuccinate synthase by 4-fold and significantly downregulates ADA in wild *C. militaris* [177,178], suggesting that this factor has a positive effect on COR biosynthesis. Thus, CmCRY-DASH, required for FB-CM development, impairs COR biosynthesis in a Cmwc-1-interdependent manner [179]. In addition, in *C. militaris*, the production of COR is markedly increased under heat stress conditions compared with normal conditions as a result of the upregulation of *CCM_04437*, which encodes a metal-dependent phosphohydrolase, and *CCM_04438*, which encodes adenosine-triphosphate phosphoribosyl- transferase [172].

### 7.4. Metabolic Mechanisms of COR in C. militaris and O. sinensis

COR biosynthetic mechanism: The biosynthetic mechanism of COR involved in purine biosynthesis has attracted broad interest in the past seven decades. Previous work indicated that adenine [180] and adenosine [181] are the direct precursors of COR biosynthesis and that the biosynthesis of COR may resemble that of 2′-deoxynucleotides [182]. Based on the whole genome sequence of *C. militaris* [183], COR is speculated to be produced through both de novo synthesis and a salvage pathway. In studies that examined COR biosynthesis in *C. militaris*, adenylosuccinate synthase, encoded by iron-induced *purA*, induced the formation of inosine monophosphate (IMP) which increased the level of AMP, and COR is subsequently produced from AMP [184]. Similar work demonstrated that COR may be formed from adenosine and its derivatives (AMP and adenosine-diphosphate) during purine biosynthesis in *C. militaris* [185]. In addition, COR may also be generated from ribonucleotides in a reaction catalyzed by ribonucleotide reductases (RNRs) in species of *Cordyceps* [186,187]. Thus, RNRs specifically catalyze the transformation of nucleotides to 2′-deoxynucleotides rather than the transformation of nucleotides to 3′-deoxynucleotides [188]. Previous studies that investigated *O. sinensis* found that adenosine may be phosphorylated by adenosine kinase before reduction by RNRs in the fruiting body [157]. As a result of the comparative analysis of *C. militaris* and *O. sinensis*, it is speculated that COR is most likely produced via the phosphorylation of adenosine prior to the unclear reduction process. Notably, the biosynthesis of COR was completely described by comparing the conserved genes involved in the COR biosynthetic pathway of two COR-producing and distantly related fungi (*A. nidulans* [25] and *C. militaris*). Mechanistically, the cns1 and cns2 enzymes cocatalyze COR biosynthesis. The Cns3 enzyme first catalyzes the reaction that changes adenosine into adenosine-3′-monophosphate (3′AMP). Next, 2′-carbonyl-3′-deoxyadenosine (2′-C-3′-dA) is generated through the dephosphorylation of 3′-AMP by Cns2. Finally, COR is produced from 2′-C-3′-dA by Cns1 [24].

The cellular detoxification of COR in *C. militaris*: Several potential pathways contribute to the cellular detoxification of COR, including deamination by ADA, decreased intracellular PTN, removal of PTN/COR out by transporters, and conversion of COR. Three out of the eight putative purine deaminases encoded by genes in *C. militaris* (*CCM_07169*, *CCM_09449* and *CCM_02911*) are similar to SanADA3 [189]. Deaminases encoded by genes in *C. militaris* (*CCM_09449* and *CCM_02911*) resemble human liver ADA1 [24], which can efficiently deaminate COR in vitro in C57BL/6 mouse erythrocytes [190]. Thus, the ADA that targets COR in *C. militaris* remain unknown. In addition, PTN, which is transported by the adenosine-triphosphate-binding cassette (ABC) transporter Cns4 and is expressed only in the FB-CM, not the mycelia, can prevent the deamination of COR by ADA through a Cns3-mediated process [24]. In most cases, drug transporters pump superabundant toxic metabolites, such as COR and PTN, out of cells to decrease cellular detoxification; however, the identity of the COR transporter remains unclear. In addition, toxic COR may be converted to other components, such as COR-TP. Thus, few studies have revealed the mechanisms of COR conversion.

### 7.5. Comparative Analysis of the COR-Associated Mechanisms Involved in the Fruiting Body of C. militaris and O. sinensis

Signal transduction in the fruiting body: Pheromone receptors, such as GPCRs, regulate fungal fruiting-body formation. It is speculated that GPCR-mediated signaling pathways play an important role in fruiting-body development. The cAMP-dependent protein kinase A (PKA) and MAPK pathways, as well as many transcription factors (C2H2 zinic fingers), are involved in the fruiting of *O. sinensis*; however, GPCR signaling are not involved [157]. In the development of the fruiting-body of *C. militaris*, signal transduction is markedly more active in FB-CM than mycelia [191], suggesting a possible relationship between the COR metabolic pathway and signal transduction. GPCRs, MAPK and cAMP-dependent PKA signaling and the major transcription factor (Zn2Cys6) all exist in the fruiting body of *C. militaris*. Notably, the level of GPCRs significantly increased during initial fruiting, and MAPK signaling plays a more important role than PKA in FB-CM development [183]. Considering that the *O. sinensis* fungus retains a rather low COR-producing capacity while the fruiting body of *C. militaris* can produce abundant COR, comparing the differential signaling involved in the fruiting development between *O. sinensis* and *C. militaris*, GPCRs are expressed only in the fruiting body of *C. militaris*, suggesting a potential relationship between COR metabolism and GPCRs. It is speculated that COR may be biosynthesized at the initial fruiting phase and transported into the extracellular matrix. Then, COR functions through an extracellular signaling may induce GPCRs and associated cascade reaction in initial fruiting of *C. militaris*, which in turn regulates COR metabolism. In other words, GPCR signaling pathways may play vital roles in COR metabolism. This hypothesis requires further verification.

Gene expression during fruiting: A large amount of COR is produced during fruiting, and the COR content in the fruiting body significantly exceeds that in mycelia. COR, as a bioactive component with multiple functions, may be produced to help fungi survive disadvantageous factors (infection and environmental extremes) and to form fruiting bodies. The gene expression levels of two species are markedly different during fruiting. The number of protein-coding genes (9684) in FB-CM [183] is larger than that (7939) in the fruiting body of *O. sinensis* [176]. The number of peroxidase genesis significantly exceeded in *O. sinensis* compared with *C. militaris*, suggesting a higher ROS-scavenging ability in *O. sinensis*. Additionally, haloperoxidase (heme) accounts for the most abundant among peroxidase genes (rough 16.67%) in *O. sinensis*. Although the amount of proteases in *O. sinensis* is fewer than that in *C. militaris*, a signal peptide, implicated in pathogen-host interactions, in *O. sinensis* obviously exceeds that in *C. militaris*, indicating a stronger interactions between pathogen and host in *O. sinensis* [176]. Mechanistically, after insect hosts are infected by fungi, the insects produce large amounts of ROS to kill the pathogenic fungi that simultaneously develop ROS-scavenging capacity [192], for example, by increasing peroxidase expression in *O. sinensis* and by promoting COR biosynthesis in both fungi. It is speculated that the *C. militaris* fungus may rapidly produce many COR molecules to defend against unfavorable factors caused by insect hosts. In contrast, although the *O. sinensis* fungus retains a COR-producing capacity, trace amounts of COR can not efficiently suppress insect-induced injury, which directly results in increased ROS-scavenging peroxidases and pathogen-host interactions. 

Fruiting of *O. sinensis*: *O. sinensis* was successfully obtained through artificial cultivation by Sunshine Lake Pharma Co. Ltd. (Guangdong, China) [193]. Phylogenetic analysis revealed *O. sinensis* and host insects originate at the same time and in similar geographic regions, southern Tibet/Yunnan, China, suggesting a strong coevolution between host and parasite [194,195]. However, the coevolution mechanisms remain unclear. Therefore, the following two issues still exist and need further investigation: (i) the genetic coevolution of *O. sinensis* and host insects; (ii) the relationship between COR metabolism and fruiting-body formation.

## 8. Summary and Outlook

Medicinal fungi produce a variety of bioactive metabolites which have medicinal potential for treating human diseases. These natural metabolites, such as COR, are more readily accepted as therapy by people and easy to obtain. Natural products of secondary metabolites that have the potential to treat human diseases have been extensively reviewed [196]. The challenge is to investigate the fermentation conditions and mutant strains for large-scale production. In addition, new natural products derived from medicinal fungi require further investigation. Recent technologies have been developed to enable the discovery of new drugs, including comparative metabolomics technologies [197]. Considering the possible toxicity, resistance and efficiency of these potential drugs, designed derivatives of natural products, such as ADA-resistant and low-toxicity COR, as well as organ-targeted nanoparticles for COR delivery in vivo therapy, are also needed. Notably, a high-throughput and efficient nanomole-scale system for synthesizing analogs of drugs has been developed. The nanomole-scale system can simultaneously rank the affinity between analogs and targets without the need for purification via mass spectrometry [198]. 

To date, although the mechanism of COR biosynthesis in *C. militaris* has been completely described, some topics require further investigation in *C. militaris*, such as the COR transport mechanism of COR, ADA that deaminates COR, gene function of cns4, natural and mutant strains that produce high levels of COR, GPCR signaling in COR metabolic pathway and effect of COR on fruiting development. 

The heterogeneity of cancer cells has historically represented a challenge for cancer research. To address this problem, single-cell analysis technologies have been developed in recent years. The development of single-cell analysis technologies based on single-cell isolation methods will remove the limitations in traditional technologies, such as single-cell sequencing technology in cancer research [199] and gene regulation [200]. Intriguingly, a new single-cell RNA-sequencing technology, which cost only $0.01 per cell and requires basic laboratory conditions, was developed [201]. Furthermore, the ability of single-cell imaging technologies to investigate the actions of drugs has been systematically reviewed [202]. Recently, a sensitive, specific, efficient fluorescent acetylcholine indicator for monitoring cholinergic action in vivo or in vitro biological processes was developed [203]. We posit that future research on COR will be greatly improved using single-cell analysis technologies, suitable fluorescent indicators and imaging technologies. In brief, the goal of single–cell analysis, including single–cell sequencing, in understanding the action of COR is to overcome the issues of cellular heterogeneity from collective cell populations that can only exhibit the average level of these heterogeneous cells, and the outcome is to understand the true mechanisms of COR action in both disease’ cells and COR-producing cells.

## Figures and Tables

**Table 1:** Pros of COR in clinical applications.

Potential Applications	COR Actions
Specific inhibitor of mRNA polyadenylation [7,117] (+++)	Inhibition of PolyA formation of mRNA.
Anticancer activity(+++)	Inhibition of leukemia [7,33,35,38,41,42,46,118,119,120], thyroid cancer [31], breast cancer [32,51,57], lung cancer [9,40,50,54,121], prostate cancer [49,122], hepatocellular cancer [37,123,124], colonic cancer [34], gallbladder cancer [11,125], renal cancer [53], cervical cancer [43], glioblastoma cancer [10], oral cancer [52], brain cancer [36] and glioma [60].
Antitumor activity(++)	Inhibition of multiple myeloma [58], renal tumor [61], leydig tumor [59,64] and EBV-infected tumor growth-infected tumor growth [17].
Anti-inflammatory activity(+++)	Inhibition of human osteoarthritis [66], Kawasaki disease [39], lipopolysaccharide (LPS)-induced inflammation [65,126], asthma [67], intervertebral disc degeneration-induced inflammation [127], traumatic brain injury-induced inflammation [68], airway inflammation [128], spinal cord injury-induced inflammation [129], atopic dermatitis [69], local anesthesia (lidocaine) induced spinal cord neurotoxicity [70] and acute lung injury-induced inflammation [130].
Anti-oxidant activity(+)	Inhibition of radical-induced oxidative damage [75], tubulointerstitial fibrosis [73] and Parkinson’s disease [74].
Pathogen growth inhibition	Inhibition of *Bacillus subtilis*(+++) [76], adenovirus(++) [77,78], murine leukovirus(++) [79], murine sarcoma virus(+++) [80], Newcastle disease virus(+++) [81], human poliovirus (+++) [82], tobacco mosaic virus(+++) [83], vaccinia virus(+++) [84], Hepatitis C virus by terminating PolyA formation of D(+) RNA(+++) [85], *Clostridium paraputrificum*(+++) [86], *Clostridium perfringens*(+++) [86] and *Candida*(++) [87].
Other medicinal potential	Induction of cell death of *Plutella xylostella*(++) [89,90,91,92], chondrogenesis(++) [112] and insulin sensitivity(+++) [107].
	Inhibition of skin photoaging(+++) [95], hyperlipidemia(++) [107,108], endoplasmic reticulum-induced injury(+++) [62], fibrosis(++) [96,97], proliferation of vascular smooth muscle(++) [100], adipogenesis-induced hepatotoxicity(+++) [105], pain(+++) [101,102,103,131], depressant(+++) [109], hypoxia-induced neuronal injuries(++) [132], myocardial infarction(+++) [98], cerebral ischemia injury(+++) [99], skeletal muscle fatigue(+++) [104], bone loss(+++) [110], hyperuricemia(+++) [133].
Simple structure, but high potency	Derivatives of COR are easy to design as a result of the simple structure of COR. Previous work demonstrated that COR exhibits higher potency than zhankuic acid A, adenosine [124] and *N*(6)-(2-hydroxyethyl)adenosine [65].
Ligand	COR and COR-TP can be used as ligands in the molecular replacement experiments that solve the molecular structures of SAHase in *B. elkanii* [113] and human Poly(A) polymeraseγ [114], respectively.
RNA elongation inhibitor	COR was used as an RNA elongation inhibitor and bromine in BrUTP was used to elucidate the structure of active rRNA genes in the nucleolus [13].
Multiple targets recognized by COR	COR can recognize many medicinal targets. In addition, COR can inhibit PolyA formation, activates protein kinases [11] and stimulates protein phosphatases [10].

**Table 2:** COR production of different strains of *Cordyceps* and *Ophiocordyceps* from 2007 to 2018.

Strain	Strain ID	Yield	Mesurement		Strain Source	References
CM	(W)NBRC 9787	2500	AL	D	NITE, Japan	[161]
CM	(M)G81-3	8570	AL	D	UF, Japan	[162]
CM	(W)-	2.276	BS	E	UM-SAR, China	[163]
CM	(M)G81-3	8600	AL	D	UF, Japan	[164]
CC	(W)-	1.398	BS	E	SNJM-HB, China	[165]
CM	(W)-	1.743	BS	E	FNS and IE-UHF	[156]
CM	(W)14014	7350	AL	D	CCICC, China	[155]
CM	(M)G81-3	14300	AL	D	UF, Japan	[22]
CM	(W)-	7.04	BS	E	MDU, Taiwan	[166]
CS	(W)-	0.0068–0.029	BS	E	QH, HB and AH, china	[159]
CM	(W)BCRC 32219	1.7	AL	D	BCRC, Hsinchu, Taiwan	[167]
CM	(W)NBRC 10352-3	6200	AL	D	SU, Japan	[168]
OX	(W)HACM 001	0.0371	BS	E	XFM-HN, China	[160]
CM	(Md)KACC44455+SPNU1006	6.63	BS	E	KACC and SPNU, Korea	[169]
CM	(W)NBRC 103752	4920	AL	D	BRC, NITE, Tokyo, Japan	[170]
CK	(GS)Y9	0.7135	AS	C	SDU, China	[171]
CM	(W)CGMCC 3.16321	5.56	BS	E	Beijing, China	[172]
CM	(W)BCRC34380	3483	AL	C	Hsinchu, Taiwan	[173]
CP	(W)GZUCC 8552	5.311	AS	C	BM, Guizhou, China	[174]
CM	(W)CGMCC 3.16321	5.56	BS	E	Beijing, China	[172]
CM	(W)No.20130508	9.45	BS	E	Nanjing, China	[23]
PH	(W)Isolated strain	0.0346	AL	D	Qinghai, China	[175]

Note: CM: Cordyceps militaris. CC:Cordyceps cicadae. CS:Cordycepssinensis. OX: Ophiocordycepsxuefengensis. CK: Cordycepskyushuensis. CP: Cordycepspruinosa. PH: Paecilomyces hepialid. A: liquid fermentation. B: solid fermentation. C: mycelia. D: extracellular. E: fruiting body. L: μg/mL. S: mg/g. (W): wild strain. (M): mutant strain. (GS): genome shuffling strain. (Md): mated strain. A+B: mycelia of strain A mated with strain B. NITE: National Institute of Technology and Evaluation. BRC: Biological Research Center. UF: University of Fukui. SNJM-HB: Shennongjia Mountains, Hubei Province. FNS: Faculty of Natural Sciences. IE-UHF: Institute of Evolution, University of Haifa. CCICC: The China Center of Industrial Culture Collection. MDU: Mingdao University. QH: QinghaiProvince, China. HB: HubeiProvince, China. AH: Anhui Province, China. BCRC: Bioresource Collection and Research Center. SU: Shizuoka University. XFM-HN: Xuefeng Mountains in Hunan Province. KACC: Korean Agricultural Culture Collection. SPNU: Systems Plant Microbiology Laboratory of Pusan National University. SD: Shandong University. BM: Leigong Mountains. UM-SAR: University of Macau, Macau SAR.

## Supplementary Materials

The following are available online. Table S1: Anti-cancer potential of COR, Table S2: Anti-tumor potential of COR, Table S3: Anti-inflammatory and anti-oxidant potential of COR, Table S4: COR inhibiting polyadenylation of mRNA in pathogens, Table S5: Other medicinal value and biotechnological applications.
