# Supplementary material for: Therapeutic Potential and Biological Applications of Cordycepin and Metabolic Mechanisms in Cordycepin-Producing Fungi
Source: Molecules. 2019 Jun 14;24(12):2231. doi: 10.3390/molecules24122231 (PMC6632035; doi:10.3390/molecules24122231)
Supplement: Supplementary file 1 [file molecules-24-02231-s001.pdf]

Table S1. Anti-cancer potential of COR.

Cancer	Pathway	Mechanisms	Sample	O/I	References
leukemia	**POM**	↑COR->↑Inhibiting polyadenylation of mRNA	MEC	O	[7]
	**apoptosis**	↑COR/COR-MP->(×)cAMP->↑PKA->↑phosphorylate TdT->↑apoptosis	TdT-LCs	O	[118]
	**apoptosis**	↑COR->↑ROS->↑caspase8/9->↑caspase3/5/7->↑apoptosis	U937/THP-1	O	[38]
	**Cell circle**	↑COR->↑p21/↑p27->↓Cyclin D1/E->↑arrest in G1 phase	BCRC60176	O	[42]
	Cell circle	↑COR->↓p-Akt->↑GSK-3b->↓β-catenin->↓Cyclin(D1->↑arrest in G1 phase	U937	O	[41]
	**apoptosis**	↑COR->↑Caspase-3->↓PARP->↑apoptosis	HL-60	O	[119]
	apoptosis	↑COR->↑ROS->↑Bax(cytoplasm)->↑cytochrome c(cytoplasm)->↑apoptosis	K562/293T	O	[120]
	apoptosis /PTKs	↑COR->↓p-PI3K/↓p-Akt->↓p-hTERT->↓telomerase->↑apoptosis	U937/THP-1	O	[33]
	apoptosis	↑COR->↑p53->↑Bax(cytoplasm)>↑caspase-9->↑apoptosis	NB-4/U937	O	[35]
	**autocrine**	↑COR->↓β-catenin/↓N-cadherin->↑apoptosis	U937/K562	O/I	[46]
	**paracrine**	↑COR->↑Dkk1&&[↓NFκ B->↓VCAM-1/↓IL-8/↓IL-6/↓FAK/↓CXCL12]->↓LSCs/↓LSIs	MSSCs		
Thyroid cancer	**apoptosis**	↑COR->↑Adenosine A1 receptor/↑Adenosine A3 receptor->↑IC>↑calpain-> ↑caspase7-> ↓PARP->↑apoptosis	CGTH W-2	O	[31]
Breast cancer	Invasion/ MAPK	↑COR->↓TPA->↓AP-1->↓MMP-9->↓invasion	MCF-7	O	[51]
	**apoptosis**	↑COR->↑Bax(cytoplasm)->↑cytochrome c(cytoplasm)->↑caspases-9->↑caspases-3->↓PARP->↑apoptosis	MDA-MB-231	O	[32]
	**autophagy**	↑COR->↑LC3-II/↑AVOs **->**↑autophagic cell death	MCF-7		
	apoptosis	↑COR->↓PARP->↓repair of DNA single strand->↓highly undifferentiated cancer cell death	MDA-MB-231/MDA-MB-435	O	[57]
Lung cancer	Anti-inflammatory	↑COR->↓TNF-α->↓LPS->↓lung inflammation	Lung injury/mice	I	[121]
	apoptosis /NFκ B	↑COR->↓PARP-1->↑apoptosis	MCF-7	O	
	**migration /MAPK**	↑COR->↓MMP-9/↓MMP-2->↓migration	CL1-0	O	[50]
	**proliferation**	↑COR->↓tyrosinases->↓proliferation	A549/ Ca alu-3	O	[54]
	apoptosis	↑COR->↓Bcl-2(cytoplasm)/↑Bax(cytoplasm)->↑C-caspases-3->↑Arrest in G_0/G_1 phase	H1975	O	[9]
	**Cell circle**	↑COR->↓p-EGFR->↓p-AKT/↓p-ERK1/2->↑Arrest in G_0/G_1 phase			
	**apoptosis**	↑COR->↓NOS->↓NO->↓p-EKR->↑GSK-3β->↓Slug->↑Bax(cytoplasm) ->↑C-caspases-3->↑apoptosis	A549	O	[40]
Prostate cancer	migration /invasion	↑COR->↓p-Akt->↓MMP-9/↓MMP-2->↑TJA/↓migration/↓invasion	LNCaP	O	[49]
	apoptosis	↑COR->↑ROS->↑Bax(cytoplasm)->↑cytochrome c(cytoplasm)-> ↑caspases-9->↑caspases-3->↓PARP/↓MMP ->↑apoptosis	PC-3	O	[122]
Hepatocellular cancer	apoptosis	↑COR->↓p-JNK->↑Bax(cytoplasm)/↓Bcl-2(cytoplasm)/↓Bcl-xL/↓IAP->↑caspase->↓PARP/↑C-PRAP /↓β-catenin->↑apoptosis	TRAIL-Hep3B	O	[123]
	**migration**	↑COR->↓ERp57/↓GLUD-1/↓GST-pi/↓PGK-1->↓migration	HepG2/Hep 3B	O	[124]
	LI	↑COR->↑NDEA->↓AST/↓ALT/↑TNF-α/↑IL-6/↑IL-1/↓SOD/↑MDA/↓HC/↑p-PI3K/↑p-Akt/↑p-mTOR/↓Nrf2/↓HO-1/↑p-IκBα/↑p-NF-κB/p65	MAM(6-8w)N	I	[37]
	LI	↑COR->IACIILIN			
colonic cancer	**apoptosis**	↑COR->↑DR3->↑caspase-8->↑caspase-1&&[↑C-caspase-3->↑C-PRAP]->↑apoptosis	HT-29	O	[34]
		↑COR->↑ROS->(×↑)p53->(×↑)Bax/(×)Bcl-2&&↑caspase-8->↑caspase-1&&[↑C-caspase-3->↑C-PRAP]->↑apoptosis			

Gallbladder Cancer	apoptosis	↑COR->↑Bax/↓Bcl-2->↑C-caspase-9->↑C-caspase-3->↑C-PARP->↑Arrest in S phase	NOZ/GBC-SD	O	[125]
	proliferation	↑COR->↑AMPK->↓mTORC1->↓MDR/↓HIF-1α->↓proliferation	GBC-SD	O	[11]
Renal cancer	proliferation	↑COR->↑p-p38 MAPK->↑p-Erk->↓proliferation	786-O	O	[53]
cervical cancer	**Cell circle**	↑COR->↓CHK1/↑p-CHK1->↓cylinB1/↓Cdc2->↑Arrest in G_2/M	HeLa	O	[43]
Glioblastoma cancer	**migration**	↑COR->↓integrin β1 receptor->↓p-FAK->↓p-paxillin->↓p-Akt ->↓migration	ANM/U87MG/LN229	O/I	[10]
Oral Cancer	AJ	↑COR->↑E-cadherin/↓N-cadherin->↓migration/Arrest in G_2/M	OCBM /SAS/OEC-M1	O/I	[52]
brain cancer	**apoptosis**	↑COR->↓GPX/↓SOD/↓Catalase->↑ROS&&↑LC3I/II/↑p53->↑Bax/↓BCL-2->↑Caspase-9->↑Caspase-3-> ↑autophagy/↑apoptosis	SH-SY5Y/ U-251	O	[36]

Note:

POM: Polyadenelation of Mrna. **MEC:** murine erythroleukemia cells. **TdT-LCs:** TdT-positive leukemia cells. **p-Akt:** phosphorylated Akt. **p-GSK-3b:** phosphorylated GSK-3b. **p-MAPK:** phosphorylated MAPK. **O:** *In vitro*(Out of body). **I:** *in vivo*(In body). **IL:** interleukin. **SOD**:Superoxide Dismutase. **NOS**:nitric oxide synthase. **GPX**:Glutathione peroxidase. **LC3-II:** an autophagosome marker and the cytoplasmic form LC3-I (18 kDa) is converted to LC3-II during autophagy[16]. **LPS:** lipopoly saccharide. **AVOs:** acidic vesicular organelles. **PLC:** phospholipase C. **EGFR:** epidermal growth factor receptor. **p-EGFR:** phosphorylated epidermal growth factor receptor. **AP-1 and NF-κB:** transcription factors, binding to the promoter of MMP-9 gene and playing an important role in regulating MMP-9[15] . **IC:** intracellular calcium. **LSCs:** leukemic stem cells. **MSCs :** mesenchymal stromal/stem cells. **MMPs:** matrix metalloproteinases, such as MMP-2 and MMP-9. **TPA:** 12-O-tetradecanoylpho-bol-13-acetate. **PARP:** Poly (ADP-ribose) polymerase. **MI:** mitochondria injury. **TJA:** tight junction activity. **CMI:** cell migration and invasion. **TRAIL:** tumor necrosis factor-related apoptosis-inducing ligand. **MAM(6-8w) N:** Male Akr mice of 6-8 weeks treating with N-nitrosodiethylamine. **NDEA:** N-nitrosodiethylamine. **ILI:** indicators of liver injury. **LI:** liver injury. **HC:** histopathology condition. **IACIILIN:** Inhibiting all the changes of indicators in the injury liver induced by NDEA. **↑:** activated or increased by cordycepin. **↓:** inactivated or decreased by cordycepin. **(×↑):** slightly increase by cordycepin. **(×↓):** slightly decrease by cordycepin. **(×):** not significantly induced/inhibited by COR or pathway didn't exist in the work. **&&:** and. **(?):** speculated pathway. **DR3:** death receptor3. **mTORC1:** mTOR complex 1(involving mTOR, Raptor and mSIN1). TJA: tight junction activity. **HIF-1α:** hypoxia-inducible factor 1α. **MDR:** multiple drug resistant. **AMPK:** AMP-activated protein kinase. **A->B:** upstream A induces down stream B. **ANM:** athymic nude mice. **OCBM:** Oral Cancer-Bearing Mice. **AJ:** Adherens Junctions. **FA:** Focal Adhesion.
AG: Antioxidant Genes.

Table S2. Anti-tumor potential of COR.

Tumor	Pathway	Mechanisms	Sample	O/I	References
multiple myeloma	**apoptosis**	↑COR->↑COR-TP->↓transcripts of *MET*->↑apoptosis	MM.1S	O	[58]
Renal	apoptosis	↑COR->↑p-elF2α->↑p-mTORC1->↓NF-κB(↓p65)->↑Sensitizing TNF-α-induced apoptosis	NRK-52E	O	[61]
Leydig Tumor	**MAPK**	↑COR->↑p-PKC->↑p-JNK/↓p-ERK1/2 -> ↑steroidogenesis /↑tumor cell death	MA-10	O	[64]
	**apoptosis**	↑COR->↑ROS->↑caspase-8->↑caspase-3->↓PARP-> apoptosis	MA-10	I/O	[59]
	**Cell circle**	↑COR->(PI3K)↓p-AKT->↓p-mTOR			
		↑COR->↑TGFβ2->↑p-p38->↑p-p53->↑p21->Arrest in G1 phase			
	**autophagy**	↑COR->↑LC3 II->↑autophagy			
glioma	apoptosis	↑COR->↑Adenosine A_{2A} receptor->↑p-p53->↑C-caspase-7->↓PARP -> ↑apoptosis	C6 glioma	O	[60]
EBV-PT	**MAPK**	↑COR&&doxorubicin->↑p-PKC->↑p-p38 ->↑p-C/EBPβ(CTF) ->↑*BZLF1* promoter->↑inhibiting EBV-infected tumor growth	SMAE	I/O	[17]
IS	**-**	↑CE-CM->↑CP/↑SP/↑NK-CA/↑cytokines(↑IFN-γ/↑TNF-α/↑IL-2/↑IL-10)->↑IS	CI-IM	I	[146]

Note:

COR-TP: COR triphosphate. **BMDMs:** bone marrow-derived macrophages. **O:** *In vitro*(Out of body). **I:** *in vivo*(In body). **SMAE:** SCID mice/AGS-EBV. **EBV-PT:** Epstein–Barr virus-positive tumor. **CTF:** cellular transcription factor. **CE-CM:** Culture extract of *C. militaris*. **IS:** Immunostimulatory. **CP:** cell proliferation. **SP:** splenocyte proliferation. **NK-CA:** NK cell activity. **CI-IM:** Cyclophosphamide-induced immunosuppressed mice. **C/EBPβ:** CCAAT/enhancer binding protein β, which can bind to *BZLF1* promoter and activates the transcription of *BZLF1*.

Table S3. Anti-inflammatory and anti-oxidant potential of COR.

Disease	Pathway	Mechanisms	Sample	O/I	References
**RIOD**	**-**	↑COR->↑SOD/↑CAT/↑GPx/↑GR/↑GST/↑GSH/↑VC/↑VE/↓MDA/↓AST/↓ALT/ ↓urea/↓creatinine->↑antioxide/↓lipid peroxidation	aged rats	I	[75]
tubulointerstitial fibrosis	ROS	↑COR->↓NADPH oxidase->↓ROS -> ↑antioxide	HK2	O	[73]
Parkinson's disease	ROS	↑COR->↓ROS/↓MDA/↑SOD/↑GSH-Px->↓6-OHDA-INT/↑antioxide	PC12	O	[74]
human osteoarthritis	NF-κB	↑COR->↓p-IκB-α->↓p65 of NF-κB(nucleus) ->[↓COX-2->↓PGE2]&&[↓iNOS->↓NO]&&[↓MMP-13/↓IL-6] ->↓Inflammation<br>↑IL-1β->↑p-IκB-α->↑p65 of NF-κB(cytoplasm→nucleus) ->↑COX-2/↑iNOS/↑MMP-13/↑IL-6 ->↑Inflammation	IHOC	O	[66]
Kawasaki disease	NF-κB	↑COR->↑p-LKB1->↑p-AMPK->↓ROS&& ↓NF-κB->↓LPS-induced TNFα->↓vasculitis	RAW 264.7/ BMDMs	O	[39]
**LPS-induced inflammation**	**NF-κB**	↑COR/↑adenosine->↓TNF-α/↓PGE2->↓LPS induced Inflammation<br>↑HEA->↓Toll-like receptor 4->↓p-IκB->↓p-p50(nucleus)/↓p-p60(nucleus)-> ↓TNF-α/↓PGE2 /↓COX-2->↓ Inflammation<br>↑LPS->↑Toll-like receptor 4 ->↑IκB-α/↑p-IκB-α->↑IL-1β/↑TNF-α/↑COX-2/↑PGE2->↑ Inflammation	RAW 264.7	O	[65]
asthma	MAPK	↑COR->↓eotaxin/↓ICAM-1/↓IL-4/↓IL-5/↓IL-13/↓airway/↓p-NF-κb/↓p-p38-MAPK->↓airway hyperreactivity	murine	I	[67]
IDD	NF-κb	↑COR->↓MD/↓MI/↓p-NF-κb->↓Inflammation	RNPC/IDO	O	[127]
TBI	-	↑TBI->↓ZO-1/↓occludin<br>↑COR->↓pro-inflammatory factors(↓IL-1β/↓Inos/↓MPO/↓MMP-9) /↑anti-inflammation-associated factors(↑arginase 1/↑IL-10)/↓NOX1->↑BBBI	Rat	I	[68]
CA	MAPK	↑COR->↓IgE/↓eosinophils/↓neutrophils/↓IL-5/↓IL-13/↓p-p38-MAPK/↓TNF-α/↑A2AR/↓TGF-β1->↓airway remodeling	Rat	I	[128]
SCI	-	↑COR-EE->↓MMP-9->↓inflammatory cytokines&&mediators(↓TNF-α, ↓IL-1β/ ↓IL-6/↓COX-2/↓iNOS)&&↓chemokines(↓Gro-α/↓Mip-2α) /↓p-p38MAPK/↓proNGF->↓DBSCB	MSDR	I	[129]
LISPIC	ROS	↑COR->↑p-c-Src->↑NADPH oxidase(↑p-p47phox subunit) ->↑ROS->↑Nrf2(nucleus)>↑HO-1 ->↓TNF-α/↓IL-6->↓Inflammation	RAW264.7	O	[126]
AD	-	↑COR->↓IL-13/IL-6/↓TNF-α/↓IL-1β->↓Inflammation	HMC-1	O	[69]
ASCN	-	↑COR->↓Caspase-9/↓MMP3->↓NILA	RDRGNs	O	[70]
ALIL	NF-κb	↑COR->↓MPO/↓MDA/↓TNF-α/↓IL-1β/↓p-p65 NF-κb/↓p-I-κB/↑Nrf2 ->↑HO-1->↓Inflammation	MBCM	I	[130]

Note:

O: *In vitro*. **I:** *in vivo*. &&: and. **RIOD:** radical-induced oxidative damage. **GSH-Px:** glutathione peroxidase. **SOD:** superoxide dismutase. **6-OHDA-INT:** 6-hydroxydopamine-induced neurotoxicity. **VC:** Vitamin C. **IL-1β:** interleukin-1beta. **IHOC:** IL-β-induced human osteoarthritis chondrocytes. **iNOS:** inducible nitric oxide synthase. **PGE2:** prostaglandin E2. **NO:** nitric oxide. **COX-2:** cyclo-oxygenase. **NF-κB:** nuclear factor kappa-B. **VE:** Vitamin E.**IkappaB-α:** the inhibitor of NF-κB in the cytoplasm. **iNOS:** inducible nitric oxide synthase. **IgE:** immunoglobulin E. **ICAM-1:** intercellular cell adhesion molecule-1. **IDD:** Intervertebral disc degeneration. **BBBI:** blood-brain barrier integrity. **MD/MI:** matrix degradation/macrophage infiltration. **ZO-1:** zonula occludens protein-1. **CA:** chronic asthma. **RNPC/IDO:** rat nucleus pulposus cell/intervertebral disc organ. **TBI:** traumatic brain injury. **RDRGNs:** rat spinal cord dorsal root ganglia neurons. **SCI:** spinal cord injury. **MSDR:** Male Sprague-Dawley rat. **MBCM:** Male BALB/c mice(6-8 weeks). **DBSCB:** disruption of blood-spinal cord barrier. **NILA:** neurotoxicity induced by local anesthesia(lidocaine). **HO-1:** heme oxygenase-1. **COR-EE:** cordycepin-enriched extract from *Cordyceps militaris*. **LISPIC:** LPS-induced secretion of proinflammatory cytokines. **ALIL:** acute lung injury induced by LPS. **ASCN:** local anesthesia(lidocaine) induced spinal cord neurotoxicity. **AD:** Atopic dermatitis. **Complex of Nrf2-Keap1:** the complex is present in the cytoplasm under normal conditions, thus, Keap1 is degraded via ubiquitination under stimuli(such as ROS) and Nrf2 then moves from the cytoplasm to the nucleus and binds related genes to regulate transcription.

Table S4. COR inhibiting polyadenylation of mRNA in pathogens.

Ogenic Microorganism	Type	In vivo/In vitro	References
Bacillus subtilis	bacteria	In vitro	[76]
adenovirus	virus	In vitro	[77]
			[78]
murine leukovirus	virus	In vitro	[79]
murine sarcoma virus	virus	In vitro	[80]
Newcastle disease virus	virus	In vitro	[81]
human rhino/poliovirus	virus	In vitro	[82]
tobacco mosaic virus	virus	In vitro	[83]
vaccinia virus	virus	In vitro	[84]
Hepatitis C virus	virus	In vitro	[85]
Clostridium paraputrificum	bacteria	In vitro	[86]
Clostridium perfringens			
Candida	fungus	in in vivo kidneys/liver/spleen	[87]

Table S5. Other medicinal value and biotechnological applications.

Bioactivities	Mechanisms	I/O	References
insecticidal activities	↑COR->direct effect on *Plutella xylostella* rather than inhibiting chitin synthesis	I	[89]
	↑COR->Inducing programmed cell death of *Trypanosoma brucei*	I	[90]
	↑COR&&↑pentostatin->↑induced curative death of *Trypanosoma evansi in vivo* mice	I	[91]
	Combination of COR(2mg/kg)&&Pentostatin(0.2mg/kg) was efficient and low toxic therapy for mice infected with *Trypanosoma evansi*	I	[92]
anti-skin photoaging	↑COR->↑p-IκB-α(cytoplasm)->↑p50 NF-κB (nucleus)/↑p65 NF-κB (nucleus) ->↓MMP-1/↓MMP-3	O	[95]
Preventing hyperlipidemia	(*In vivo*) ↑COR->↓TC/↓TG/↓LDL-c	I/O	[107]
	(*In vitro*) ↑COR->↑p-ACC/↑p-AMPK		
MUPR/ERI	↑COR->Adenosine A3 receptor/↑p-eIF2α/↓ERI(transcription)->↓ATF4 ->[↓CHOP]&&[↓GADD34->↑S-p-eIF2α]-> ↓ERA	I/O	[62]
Inhibiting fibrosis	↑COR->IRE1->ASK1->↓p-JUK->↓ERA	O	[96]
	↑COR->↑E-cadherin/↓vimentin->↑Inhibiting lung fibrosis		
	↑TGFβ1->↓E-cadherin/↑vimentin		
	↑COR->↓CAGA box/↓BRE/↓p-Smad1/2/3/↑p-eIF2α->↑inhibiting kidney fibrosis	I/O	[97]
	↑TGFβ1/↑BMP-4->↑BRE/↑p-Smad1/2/3		
IPVSM	↑COR->↑Ras-GTP->↑p-ERK1/2->↑p27KIP1(CDK inhibitor)->↓CDK2- cyclin E /↓CDK4-cyclin D1 -> Arrest in G1 phase	O	[100]
Inhibiting hepatotoxicity	(adenosine transporter)↑COR->(insulin receptor)↓p-PKB/↑p-AMPK->↓mTORC1->↓C/EBPβ-> ↓PPARγ->↑Inhibiting adipogenesis	O	[105]
	↑CECM->↓AST/↓ALT/↑ADH/↑ALDH/↓LD->↓alcoholic-induced hepatotoxicity	I	[106]
Effect on nervous system	↑COR->disrupting the translation in the nociceptor->↓chronic pain	I	[103]
	↑COR->↓fEPSPs /↓AMPA-RMR/↓NMDA-RMR/↓FMSEPC ->↑Inhibiting excitatory synaptic transmission	O	[18]
	↑glutamate->(×)caspase 12/↑ROS/↑Ca2+ influxes->↑oxidative and ER stress cell death	O	[19]
	↑COR->Adenosine A1 receptor->↓ROS/↓Ca^{2+} influxes->↑Bax ->↓MAPKs(JNK/ERK/p38)/↓CHOP->↓glutamate induced cell apoptosis and promoting neuroprotective effects		
	↑COR->↓hyperalgesia in the peripheral nociceptors	I	[102]
	↑COR->↓CNAP'transduction Via L-type Ca^{2+} channel	O	[101]
	↑COR->↓PGE2->↓chronic pain	I	[131]
	↑COR->↑p-GluR1 S845/↑GluR1(↑AMPA-RMR)->↑Antidepressant Effect	I	[109]
	↑COR->↓A-VGSC-Cs/↑TI-NaI-R->↑easily close VGSC/↑prolong transision from inactive VGSC to closed VGSC->↓hypoxia-induced neuronal injuries	O	[132]
I/R injury	↑COR->[↑IVDP]&&[↑p-Akt/↑p-GSK-3β/↑p-p70S6K]&&[↑Bcl-2/↓Bax/↓C-caspase-3/↑HO-1] ->↓myocardial infarction	I	[98]

	(*In vivo* extracellular brain)↑COR->↓glutamate/↓aspartate	I/O	[99]
	(vitro)↑COR->↓SOD/↓MDA/↓MMP-3->↓cerebral ischemia injury		
transcription inhibitor	COR/actinomycin D as transcription inhibitors verify circadian rhythms	O	[116]
	COR as transcription inhibitor to analysis RNA decay in Plant Heat Stress Tolerance assay	O	[117]
Inhibiting hyperlipidemi a	↑COR->↑COR Interacts with γ1 subunit of AMPK->↑p-AMPK->↓lipid(intracellular)	O	[108]
Inhibiting skeletal muscle fatigue	↑COR/↑Ca2+ influxes(Ca2+ channel-dependent pathway)->↑MAMCF/inhibiting DMCF-RS/↑RARMC->↓skeletal muscle fatigue	O	[104]
Effect on bone	↑COR->[↑Sox9/↑collagen type II]&&[↓Nrf2]->↑chondrogenesis	O	[112]
	↑COR->[↓PI3K->↑Bapx1]&&[Notch1/2->NICD->↓Hey1/↓Runx2->↓collagen type X/↓MMP13->↑inhibiting chondrocyte hypertrophy		
	(vitro)↑COR ->↓ROS/↑IRF8/↓NFATc1->↓Osteoclastogenesis->↑Preventing Bone Loss	I/O	[110]
	(vivo)↑COR ->↓bone loss/↑sustain bone microarchitecture/↑recovery bone mineralization		
	↑COR->↓Ethanol-induced osteoclastogenesis /↑β-catenin/↑Runx2->↑Protecting FHOA	I/O	[111]
Antiplatelet	↑CECM->↓thromboxane A2 synthase/↓arachidonic acid/↓p-p38-MAPK/↓p-ERK2->↓thromboxane A2->↑inhibiting platelet induced by collagen and ADP	I/O	[20]
Studys of molecular structure	COR, as a ligand, is used in the molecular replacement experiments that are used to identify SAHase in *Bradyrhizobium elkanii*	O	[113]
	COR is used as a RNA elongation inhibitor to control the transcriptional reaction rate.	O	[13]
Anti-hyperuricemia	↑COR->↓uric acid transporter 1(kidney)->↓serum uric acid without affecting liver/renal/spleen functions	I	[133]
radiolabel	3′-end radiolabeled with [α-32P]-COR-TP	O	[21]

Note:

&&: and. TC: total cholesterol. **TG:** Triglycerides. **LDL-c:** low-density lipoprotein cholesterol. **p-ACC:** phospho-acetyl-CoA carboxylase. **SOD:** superoxide dismutase. **MDA:** malondialdehyde. **PERK:** PKR-like ER kinase. **(×):** did not affect the target. **MUPR/ERI:** modulating unfolded protein response against endoplasmic reticulum stress-induced cell injury. **LD:** lipid droplet. **ERA:** endoplasmic reticulum stress-induced apoptosis. **ATF4:** transcription factor 4. **BMP-4:** bone morphogenetic protein-4. **S-p-eIF2α:** sustained phosphorylation of eIF2α. **[A]&&[B]:** involve pathways of A and B. **TGFβ1:** transforming growth factor beta-1. **BRE:** bone morphogenetic protein-responsive element. **IPVSM:** Inhibiting proliferation of vascular smooth muscle. **CECM:** cordycepin-enriched *Cordyceps militaris*. **ADH:** alcohol dehydrogenase. **ALDH:** acetaldehyde dehydrogenase. **fEPSPs:** amplitudes of field excitatory postsynaptic potentials. **NMDA-RMR:** N-methyl-d-aspartic acid receptor-mediated responses. **AMPA-RMR:** alpha-amino-3-hydroxy-5-methyl-4-isoxazole-propionic acid receptor-mediated responses, such as GluR1 and GluR2. **FMSEPC:** frequency of miniature spontaneous excitatory postsynaptic currents. **CNAP:** compound nerve action potential. **PGE2:** prostaglandin-E2. **PFC:** prefrontal cortex. **HIP:** hippocampus. **TI-NaI-R:** time of inactive Na+ influx recovery. **A-VGSC-Cs:** amplitude of voltage-gated sodium channel currents. **NaI:** Na+ influx. **VGSC:** voltage-gated sodium channel. **IVDP:** left ventricular developed pressure. **HO-1:** heme oxygenase. **SMC:** skeletal muscle contraction. **I/R:** ischemic/reperfusion. **MAMCF:** The max amplitude of muscle contractile force. **DMCF-RS:** the decrease of muscle contractile force via repetitive stimulation.

RARMC: recovery amplitude and ratio of muscle contraction. **NFATc1:** nuclear factor of activated T cells c1. **IRF8:** interferon regulatory factor 8. **FHOA:** femoral head's osteonecrosis induced by Alcohol. **SAHase:** S-Adenosyl-l-homocysteine hydrolase.

O: *In vitro*(Out of body). **I:** *In vivo*(In body).

Note: The reference numbers in the supplementary tables are the same as those in the body of the paper.
